# Free Functional Gracilis Muscle Transfer for Severe Pediatric Volkmann Ischemic Contracture After Traditional Splinting of a Supracondylar Humerus Fracture: A Case Report

**DOI:** 10.7759/cureus.112777

**Published:** 2026-07-16

**Authors:** Mohammed Benlili, Doha Arreyouchi, Ayat Allah Oufkir

**Affiliations:** 1 Plastic and Reconstructive Surgery, Faculty of Medicine and Pharmacy of Oujda, Mohammed First University, Oujda, MAR

**Keywords:** free functional muscle transfer, gracilis flap, microsurgery, pediatric hand reconstruction, supracondylar humerus fracture, volkmann ischemic contracture

## Abstract

Volkmann ischemic contracture is a severe and largely preventable complication of untreated or delayed compartment syndrome. In children, it is most commonly associated with supracondylar humerus fractures, especially when external compression from casts or splints is not promptly recognized. We report the case of a six-and-a-half-year-old right-handed girl who developed severe left upper-limb dysfunction after a closed supracondylar humerus fracture treated in a rural setting with tight traditional splint immobilization, locally known as “Jbira.” No fasciotomy was performed during the acute phase. She presented 18 months after the injury with elbow stiffness, fixed forearm pronation, wrist stiffness, proximal interphalangeal joint deformities, loss of active digital flexion, and paresthesia involving the thumb and index finger. The condition was classified as severe Volkmann ischemic contracture. A staged reconstruction was performed. The first stage included release of fibrotic forearm tissue, excision of nonfunctional flexor fibrosis, ulnar nerve neurolysis and transposition, median nerve reconstruction with a sural nerve graft, passive joint mobilization, and radial derotational osteotomy. One year later, because active finger flexion remained insufficient, a free functional innervated gracilis muscle transfer was performed. At 12-month follow-up after the gracilis transfer, the patient achieved Medical Research Council grade M3 active finger flexion, partial fist formation, and functional grasp sufficient to hold a glass and a bottle. This case supports staged free functional gracilis transfer as a useful salvage option in severe pediatric Volkmann ischemic contracture.

## Introduction

Volkmann ischemic contracture is the consequence of irreversible ischemic injury to the muscles and nerves of the forearm following untreated or inadequately treated compartment syndrome. In children, it is classically associated with supracondylar fractures of the humerus, particularly when diagnosis is delayed or when external compression from casts, dressings, or splints contributes to increased compartment pressure [1].

Established Volkmann ischemic contracture may combine muscle fibrosis, joint stiffness, nerve dysfunction, deformity, and severe impairment of hand function. The Tsuge classification divides established cases into mild, moderate, and severe forms according to the extent of muscle necrosis and nerve involvement [2]. In severe forms, the flexor muscle mass is extensively degenerated, and conventional tendon transfers may be insufficient because the native forearm muscles are no longer functional.

In such cases, reconstruction is often staged and may include the release of fibrosis, neurolysis or nerve reconstruction, restoration of passive mobility, correction of skeletal deformity, and secondary free functional muscle transfer [3,4].

Free functional gracilis muscle transfer is based on the transplantation of a vascularized muscle with microsurgical arterial and venous anastomoses and coaptation of its motor nerve to a suitable recipient motor nerve. In severe Volkmann ischemic contracture, the transferred gracilis muscle can serve as a new functional motor unit when the native forearm flexor muscles are irreversibly fibrotic and unable to generate useful finger flexion. The gracilis muscle is commonly selected because it has a reliable vascular pedicle, a suitable motor nerve, adequate excursion for digital flexion, expendability, and generally limited donor-site morbidity [4].

The objective of this case report is to describe the clinical presentation, severity classification, staged surgical management, rehabilitation, and functional outcome of a child with severe established Volkmann ischemic contracture following traditional tight splinting of a supracondylar humerus fracture. We aimed to show that, in a delayed and severe pediatric presentation with irreversible flexor muscle fibrosis, a staged approach aimed first at releasing fibrosis, improving passive mobility, and addressing nerve and skeletal deformities, followed by free functional innervated gracilis muscle transfer, can provide meaningful recovery of active digital flexion. The novelty and educational value of this case lie in the association between a preventable rural immobilization-related complication and a complex staged microsurgical salvage strategy that achieved useful grasp in a child despite long-standing severe contracture.

## Case presentation

A six-and-a-half-year-old right-handed girl was referred to our department for severe functional impairment of the left upper limb secondary to Volkmann ischemic contracture. The initial injury was a closed supracondylar fracture of the left humerus that had occurred 18 months earlier in a rural setting. The fracture had been treated with tight traditional splint immobilization, locally known as “Jbira,” applied by non-medical practitioners. No fasciotomy was performed during the acute phase.

Forty-eight hours after immobilization, the patient developed progressive pain in the forearm, wrist, and hand, associated with edema and sensory-motor disturbances of the distal extremity. The splint was removed by the family after 10 days. She subsequently developed a soft-tissue defect on the proximal anterior aspect of the left forearm, probably related to excessive pressure and soft-tissue tension caused by the tight immobilization. A chronological summary of the clinical course is presented in Table 1.

**Table 1:** Timeline of clinical events Day 0 corresponds to the day of the initial injury and immobilization. Subsequent time points indicate the time elapsed from the initial injury. MRC: Medical Research Council.

Time from initial injury	Clinical event
Day 0	Closed supracondylar fracture of the left humerus in a rural setting
Day 0	Tight traditional splint immobilization, locally known as “Jbira,” applied by non-medical practitioners
Day 2	Progressive forearm, wrist, and hand pain with edema and sensory-motor disturbances
Day 10	Splint removed by the family
Day 10 onward	Proximal anterior forearm soft-tissue defect and progressive contracture
18 months	Referral with established severe Volkmann ischemic contracture
18 months	First-stage reconstruction: release of fibrosis, excision of nonfunctional flexor fibrosis, ulnar nerve neurolysis and transposition, median nerve reconstruction with sural nerve graft, passive arthrolysis, and radial derotational osteotomy
30 months	Second-stage reconstruction: free functional innervated gracilis muscle transfer
33 months	First perceptible active contraction of the transferred gracilis muscle
42 months	Twelve-month follow-up after gracilis transfer: MRC grade M3 active finger flexion and improved grasping function

On admission, the patient had a scar on the proximal anterior aspect of the left forearm, elbow flexion contracture, fixed pronation of the forearm, wrist stiffness, and flexion deformity of the proximal interphalangeal joints, mainly involving the four ulnar fingers. Mild amyotrophy of the left forearm was noted. Vascular examination showed warm distal extremities with present and symmetrical pulses.

**Figure 1:**
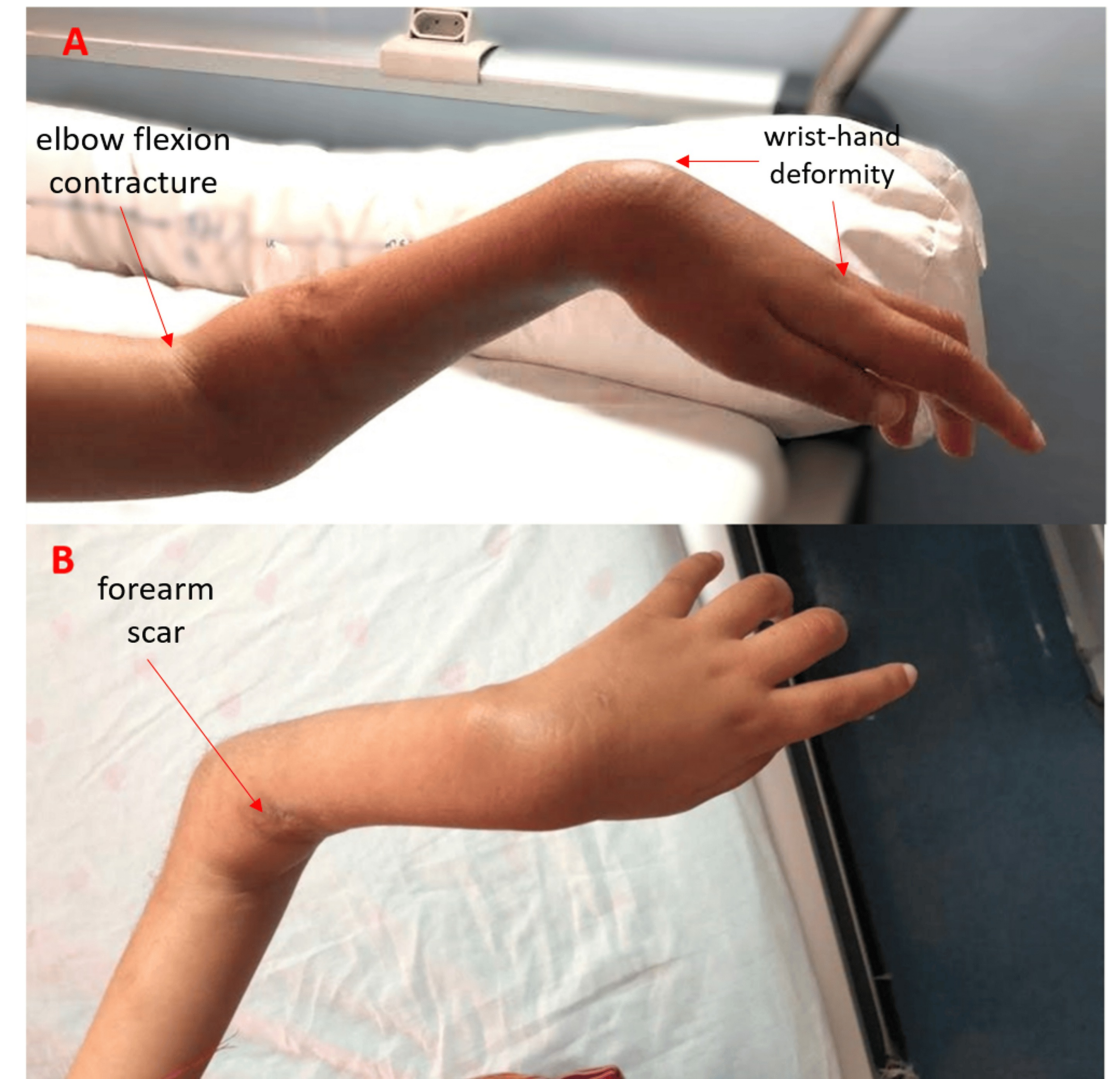
Preoperative clinical appearance of the left upper limb. (A) Lateral view showing severe elbow flexion contracture, fixed forearm pronation, wrist deformity, and digital stiffness secondary to established Volkmann ischemic contracture. The arrow highlights the elbow flexion contracture and wrist-hand deformity. (B) Anterolateral view demonstrating proximal forearm scarring, forearm amyotrophy, wrist stiffness, and flexion deformity of the fingers before staged reconstructive surgery. The arrow highlights the proximal forearm scar.

Neurological examination revealed paresthesia involving the thumb and index finger. Motor examination showed severe stiffness of the elbow, wrist, and fingers, with loss of active wrist extension, absent useful active flexion of the proximal interphalangeal joints of the four ulnar fingers, and absent thumb opposition, flexion, and extension. The preoperative clinical appearance is shown in Figure 1.

According to the Tsuge classification, the patient was classified as having severe Volkmann ischemic contracture because of extensive flexor muscle fibrosis, major loss of active digital flexion, median nerve involvement, severe joint stiffness, and marked functional impairment.

Because of the severity of the contracture, a staged reconstructive strategy was planned. The first surgical stage was performed 18 months after the initial trauma. Through a zigzag incision extending from the medial epicondyle to the wrist, dense fibrotic tissue of the forearm was released. The severely fibrotic and nonfunctional flexor muscles were divided, and the central muscular fibrosis was excised. Neurolysis of the ulnar nerve was performed, followed by subcutaneous transposition. The median nerve was found embedded in dense fibrosis and was reconstructed using a 17-cm sural nerve graft with epiperineural microsurgical suturing. The anterior interosseous nerve was fibrotic but preserved.

**Figure 2:**
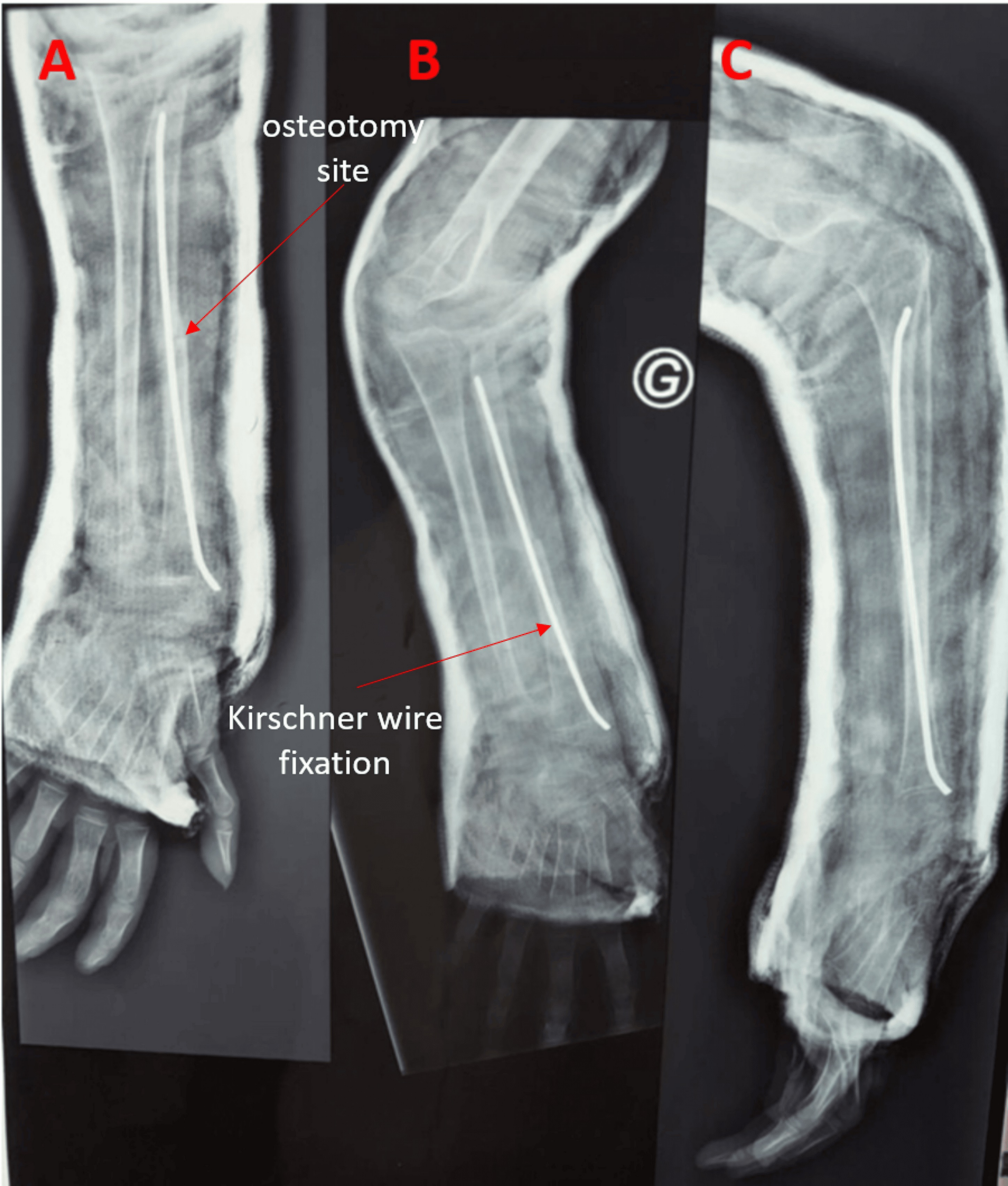
Postoperative radiographs after radial derotational osteotomy of the left forearm. (A) Anteroposterior view showing radial osteotomy fixed with a Kirschner wire. (B) Oblique view showing the corrected forearm position under immobilization. (C) Lateral view including the elbow and forearm, showing maintained alignment after osteotomy and Kirschner-wire fixation. The arrows indicate the osteotomy site and Kirschner wire fixation.

Manual arthrolysis allowed restoration of passive extension of the wrist and fingers. During the same procedure, the pediatric orthopedic team performed a radial derotational osteotomy to improve forearm supination, fixed with a Kirschner wire. Postoperative radiographs are shown in Figure 2.

Postoperatively, the limb was immobilized in an above-elbow cast, and supervised rehabilitation was started during the first postoperative week. At one-year follow-up after the first stage, sensory recovery had partially improved, with preserved nociceptive sensation and a positive Tinel sign along the median nerve pathway. However, active motor function remained severely impaired, with persistent elbow and wrist stiffness, absent active wrist extension, poor thumb opposition, and insufficient active finger flexion.

Because passive mobility and sensory recovery had improved but active digital flexion remained inadequate, a second-stage free functional innervated gracilis muscle transfer was indicated. Preoperative Doppler assessment was performed before microsurgery to evaluate the recipient vessels.

During the second operation, the recipient site was approached through an incision along the ulnar border of the left forearm. The previous median nerve grafts were identified and released. The vascular axis was explored, and neurolysis of the ulnar nerve was performed. Intraoperative neurostimulation was used to identify suitable motor fascicles of the ulnar nerve. Three motor fascicles were selected for coaptation.

A free gracilis muscle flap was harvested from the left thigh with a 16×7 cm skin paddle and a 20-cm muscle length. The vascular pedicle and motor nerve were dissected, and the donor site was closed over a suction drain. After transfer to the forearm, an end-to-side arterial anastomosis was performed to the ulnar artery, and an end-to-end venous anastomosis was performed to a vena comitans of the radial artery. The ischemia time was 54 minutes.

The motor nerve of the gracilis was coapted to the three selected motor fascicles of the ulnar nerve, and the nerve coaptation was protected using a vein conduit. This selective fascicular coaptation did not result in postoperative worsening of ulnar motor or sensory function. The muscle was fixed proximally to the medial epicondyle, and its distal tendon was sutured to the flexor digitorum profundus tendons while maintaining physiological muscle tension. The transferred muscle was well perfused at the end of the procedure. 

The limb was immobilized in an above-elbow splint with the elbow flexed at 90°, the wrist in neutral position, and the fingers in full extension. The immediate postoperative course was marked by a small area of superficial skin necrosis measuring 1×2.5 cm, which was managed by surgical debridement followed by directed wound healing. No vascular thrombosis or flap loss occurred.

The above-elbow splint was maintained for six weeks and was then replaced by a removable below-elbow dorsal splint. Rehabilitation was continued progressively, initially focusing on protection of the transfer and maintenance of passive joint mobility. Galvanic stimulation was applied through the skin paddle twice daily until the first perceptible active contraction of the transferred muscle was observed at approximately three months. Progressive strengthening exercises and active motor re-education were then initiated.

**Figure 3:**
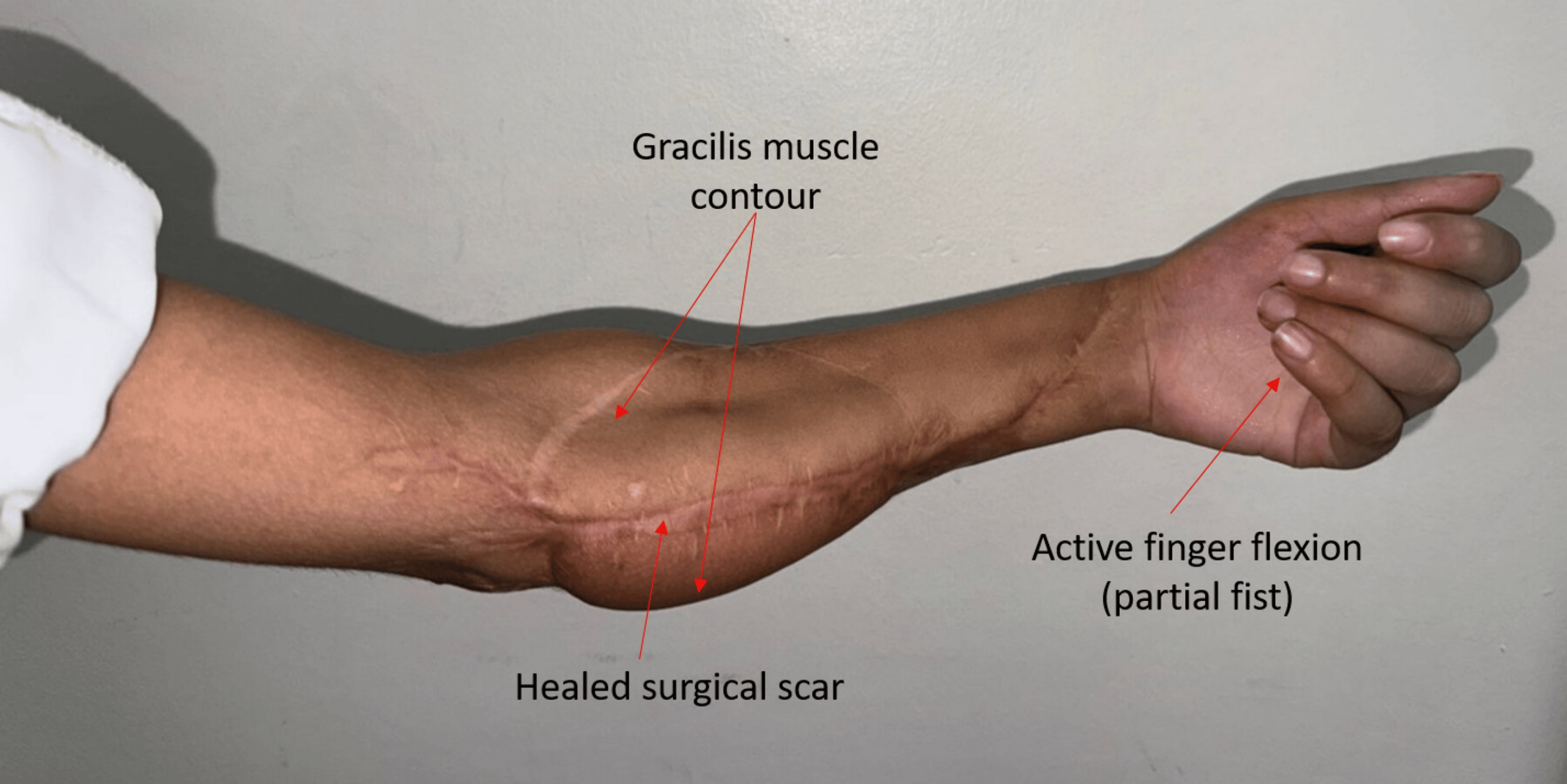
Postoperative clinical appearance after free functional gracilis muscle transfer. Clinical appearance at 12-month follow-up showing a viable transferred gracilis muscle with an acceptable forearm contour, healed surgical scars, and active finger flexion allowing partial fist formation. The patient achieved meaningful functional improvement, with Medical Research Council grade M3 active digital flexion and the ability to grasp simple objects. The arrows highlight the transferred gracilis muscle contour and active digital flexion.

At 12-month follow-up after the free functional gracilis transfer, the transferred muscle was viable, with an acceptable forearm contour and meaningful functional improvement. Active finger flexion was graded M3 according to the Medical Research Council (MRC) scale. The patient achieved partial fist formation and was able to use the hand for simple grasping activities, including holding a glass and a bottle. Passive finger flexion was complete, while active proximal interphalangeal flexion remained incomplete but clearly improved. Elbow mobility remained limited, with flexion reaching 110° and a residual extension deficit of approximately 20°. Wrist flexion was complete, but active wrist extension remained absent. No significant donor-site morbidity was observed. The functional result is shown in Figure 3 and summarized in Table 2.

**Table 2:** Functional assessment before and after staged reconstruction MRC: Medical Research Council; PIP: physical inertial poser.

Parameter	Before first-stage reconstruction	Before gracilis transfer	Twelve months after gracilis transfer
Passive finger mobility	Severely limited	Improved after release and arthrolysis	Complete passive finger flexion
Active finger flexion	Absent useful active flexion	Insufficient active flexion	MRC grade M3 active flexion
Fist formation	Impossible	Very limited	Partial fist formation
Functional grasp	Not useful	Very limited	Able to hold a glass and a bottle
Elbow range of motion	Severe stiffness	Persistent stiffness	Flexion to 110° with residual extension deficit of approximately 20°
Wrist flexion	Stiff	Improved	Complete
Active wrist extension	Absent	Absent	Absent
PIP motion	Flexion deformity and absent useful active flexion	Limited active flexion	Active flexion incomplete, but improved
Sensory status	Thumb and index paresthesia	Partial recovery with positive Tinel sign	No postoperative ulnar sensory worsening
Donor site	Not applicable	Not applicable	No significant donor-site morbidity

As this was a single-patient case report, formal inclusion and exclusion criteria and statistical analysis were not applicable. Outcome assessment was based on serial clinical examinations, passive and active range of motion, MRC grading of active digital flexion, partial fist formation, simple grasping ability, flap viability, and donor-site morbidity. The follow-up duration after free functional gracilis muscle transfer was 12 months.

Patient consent

Written and signed informed consent was obtained from the patient’s legal guardian for publication of the case details and accompanying clinical images, including the disclosure of identifiable clinical images in an open-access publication. The signed consent statement has been submitted to the journal.

## Discussion

Volkmann ischemic contracture remains a devastating but preventable complication of missed or delayed compartment syndrome. In children, supracondylar fractures of the humerus are among the classic injuries associated with this condition, especially when warning symptoms such as progressive pain, swelling, paresthesia, and motor disturbance are not recognized early [1]. In the present case, tight traditional splinting probably aggravated the ischemic process, and the absence of fasciotomy during the acute phase allowed progression to established contracture.

Severity classification is important because it guides reconstruction. According to Tsuge, severe Volkmann ischemic contracture is characterized by extensive degeneration of the flexor muscle mass, possible extensor involvement, nerve impairment, and major loss of hand function [2]. Our patient met these criteria because of extensive nonfunctional flexor fibrosis, median nerve involvement requiring graft reconstruction, loss of active digital flexion, and severe functional disability.

Treatment of severe established Volkmann ischemic contracture is challenging because tendon transfers require supple joints and functional donor muscles. When the native forearm musculature is extensively fibrotic, tendon transfers alone may not restore useful finger flexion. In such cases, staged reconstruction is recommended, including excision of fibrotic muscle, neurolysis or nerve reconstruction, restoration of passive mobility, correction of skeletal deformity, and secondary free functional muscle transfer in selected patients [3,4].

In our patient, the first stage aimed to improve local conditions for later reconstruction by releasing fibrosis, decompressing the ulnar nerve, reconstructing the median nerve, restoring passive mobility, and correcting fixed pronation through radial derotational osteotomy. The second stage was indicated because passive mobility had improved but active digital flexion remained severely deficient.

The gracilis muscle is commonly used for free functional muscle transfer because of its reliable vascular pedicle, suitable motor nerve, adequate excursion, and limited donor-site morbidity. Sabapathy et al. reported that free functional gracilis transfer is a reliable option for restoring finger flexion in severe Volkmann ischemic contracture, particularly after preliminary release, neurolysis, and recovery of passive mobility [4]. This staged strategy corresponds closely to the approach used in our case.

Several technical points were important. Preoperative Doppler assessment helped identify recipient vessels. End-to-side arterial anastomosis to the ulnar artery was used to preserve distal arterial flow. The motor fascicles of the ulnar nerve were selected by intraoperative neurostimulation, allowing coaptation of the gracilis motor nerve to functional motor fascicles. This was achieved without postoperative worsening of ulnar motor or sensory function. Selective fascicular coaptation has been described as a useful strategy in functional gracilis transfer when appropriate motor fascicles are available [5].

Postoperative rehabilitation was essential. Immobilization protected the transfer during the early healing period, while progressive therapy maintained passive mobility and later promoted motor re-education. Galvanic stimulation was used until the first perceptible contraction at approximately three months, followed by strengthening exercises. Functional recovery after free muscle transfer depends on microsurgical success, appropriate muscle tensioning, stable fixation, nerve regeneration, cortical re-education, and prolonged rehabilitation [6].

At 12 months, the patient achieved MRC grade M3 active finger flexion, partial fist formation, and useful grasp for simple daily activities. This result represents partial but meaningful recovery rather than complete restoration. Persistent limitations included absent active wrist extension, residual elbow stiffness, incomplete active proximal interphalangeal flexion, and incomplete thumb extension. These residual deficits are expected in severe long-standing Volkmann ischemic contracture, where muscle, nerve, joint, and soft-tissue damage coexist [7]. More broadly, free functional muscle transfer is an established reconstructive option for selected upper-extremity defects when local muscles are unavailable or nonfunctional [8].

This report has several limitations. First, it is a retrospectively reported single-patient case with no control or comparison group. Therefore, the findings cannot be generalized to all patients with severe Volkmann ischemic contracture. Second, selection bias is possible because this case represents a severe delayed presentation selected for complex staged microsurgical reconstruction. Third, preoperative electromyography and magnetic resonance imaging were not available, and decision-making was based mainly on clinical examination, intraoperative findings, passive mobility assessment, and Doppler evaluation. Fourth, objective functional measurements such as grip strength, pinch strength, validated hand function scores, DASH (Disabilities of the Arm, Shoulder, and Hand) score, Pediatric Outcomes Data Collection Instrument, or other patient-reported outcome measures were not available. Functional assessment was therefore based on serial clinical examinations, passive and active range of motion, MRC grading of active digital flexion, partial fist formation, simple grasping ability, flap viability, and donor-site morbidity. Finally, the follow-up duration after free functional gracilis muscle transfer was limited to 12 months, which restricts assessment of long-term durability, growth-related changes, and late functional evolution.

The interpretation of this result should be done with caution. This single case demonstrates the feasibility and potential clinical utility of staged reconstruction with free functional gracilis muscle transfer in a selected child with severe established Volkmann ischemic contracture, but it does not establish superiority over other reconstructive options or broad applicability to all patients. Treatment must be individualized according to the severity of muscle fibrosis, nerve involvement, passive joint mobility, skeletal deformity, availability of recipient vessels and donor nerves, rehabilitation potential, and family adherence to prolonged follow-up. Nevertheless, this case illustrates that staged reconstruction with free functional gracilis transfer can provide useful grasp in severe pediatric Volkmann ischemic contracture when native flexor muscles are irreversibly damaged.

## Conclusions

Severe pediatric Volkmann ischemic contracture after neglected supracondylar humerus fracture is a disabling condition that requires early recognition, severity classification, and individualized reconstruction. Prevention remains essential, and children treated with casts, splints, or traditional immobilization should be carefully monitored for signs of compartment syndrome.

In selected severe established cases with irreversible flexor muscle fibrosis, nerve involvement, and absent useful active finger flexion, tendon transfers may be insufficient. A staged approach combining release of fibrosis, nerve reconstruction or neurolysis, correction of skeletal deformity, restoration of passive mobility, and secondary free functional innervated gracilis muscle transfer may provide meaningful functional improvement. In this patient, MRC grade M3 active finger flexion and the ability to grasp simple objects represented partial but clinically useful recovery. However, because this is a single-patient case report with limited follow-up and no standardized functional outcome scores, these findings should be interpreted cautiously and cannot be generalized without further studies and longer-term follow-up.
